# Air pollution and emergency department visits for cardiac and respiratory conditions: a multi-city time-series analysis

**DOI:** 10.1186/1476-069X-8-25

**Published:** 2009-06-10

**Authors:** David M Stieb, Mieczyslaw Szyszkowicz, Brian H Rowe, Judith A Leech

**Affiliations:** 1 Population Studies Division, Healthy Environments and Consumer Safety Branch, Health Canada, Postal locator 4903C, 269 Laurier Ave West Ottawa, Ontario, K1A 0K9, Canada; 2 Department of Epidemiology and Community Medicine, University of Ottawa, Room 3105, 451 Smyth Road, Ottawa, Ontario, K1H 8M5, Canada; 3 Department of Emergency Medicine, University of Alberta, Room 1G1.43 WMC, University of Alberta Hospital, 8440-112th Street, Edmonton, Alberta, T6G 2B7, Canada; 4 Department of Medicine, University of Ottawa, Ottawa Hospital, Civic Campus, 1053 Carling Avenue, Ottawa, Ontario, K1Y 4E9, Canada

## Abstract

**Background:**

Relatively few studies have been conducted of the association between air pollution and emergency department (ED) visits, and most of these have been based on a small number of visits, for a limited number of health conditions and pollutants, and only daily measures of exposure and response.

**Methods:**

A time-series analysis was conducted on nearly 400,000 ED visits to 14 hospitals in seven Canadian cities during the 1990s and early 2000s. Associations were examined between carbon monoxide (CO), nitrogen dioxide (NO_2_), ozone (O_3_), sulfur dioxide (SO_2_), and particulate matter (PM_10 _and PM_2.5_), and visits for angina/myocardial infarction, heart failure, dysrhythmia/conduction disturbance, asthma, chronic obstructive pulmonary disease (COPD), and respiratory infections. Daily and 3-hourly visit counts were modeled as quasi-Poisson and analyses controlled for effects of temporal cycles, weather, day of week and holidays.

**Results:**

24-hour average concentrations of CO and NO_2 _lag 0 days exhibited the most consistent associations with cardiac conditions (2.1% (95% CI, 0.0–4.2%) and 2.6% (95% CI, 0.2–5.0%) increase in visits for myocardial infarction/angina per 0.7 ppm CO and 18.4 ppb NO_2 _respectively; 3.8% (95% CI, 0.7–6.9%) and 4.7% (95% CI, 1.2–8.4%) increase in visits for heart failure). Ozone (lag 2 days) was most consistently associated with respiratory visits (3.2% (95% CI, 0.3–6.2%), and 3.7% (95% CI, -0.5–7.9%) increases in asthma and COPD visits respectively per 18.4 ppb). Associations tended to be of greater magnitude during the warm season (April – September). In particular, the associations of PM_10 _and PM_2.5_with asthma visits were respectively nearly three- and over fourfold larger vs. all year analyses (14.4% increase in visits, 95% CI, 0.2–30.7, per 20.6 μg/m^3 ^PM_10 _and 7.6% increase in visits, 95% CI, 5.1–10.1, per 8.2 μg/m^3 ^PM_2.5_). No consistent associations were observed between three hour average pollutant concentrations and same-day three hour averages of ED visits.

**Conclusion:**

In this large multicenter analysis, daily average concentrations of CO and NO_2 _exhibited the most consistent associations with ED visits for cardiac conditions, while ozone exhibited the most consistent associations with visits for respiratory conditions. PM_10 _and PM_2.5 _were strongly associated with asthma visits during the warm season.

## Background

Numerous analyses have been conducted of the association between outdoor air pollution and mortality, as well as hospital admissions, owing to the wide availability of these data through national vital statistics and health care statistics systems [1-4]. While these studies have been central to establishing the occurrence of adverse health effects of air pollution even at the relatively low levels of exposure now observed in most industrialized countries, these health outcomes are generally believed to occur in a relatively small segment of the population in comparatively poor health. The emergency department (ED) is often the point of entry for those ultimately admitted to hospital. For many cardiac and respiratory conditions, however, less than half (and as few as 10% in the case of asthma) of those seen in the emergency department are admitted to hospital [5]. Thus ED visits reflect impacts in a broader segment of the population.

Since these data are not routinely available, fewer studies have been conducted, and of these, most have been based on a single city and a relatively small number of visits, for a limited number of health conditions and pollutants. Multi-city studies are believed to generate more stable results which are less prone to biases that may affect small studies in individual centers [6]. In addition, most studies have examined only daily counts of ED visits, even though information on time of registration in the emergency department affords the opportunity of examining associations on a shorter time scale. In this study, we set out to examine associations of a comprehensive array of respiratory and cardiac conditions with the full suite of conventional air pollutants in a large multi-center study, using both daily and 3 hour average measures of exposure and response.

## Methods

Air pollution data were obtained from the National Air Pollution Surveillance (NAPS) system, and weather data from Environment Canada's weather archive. We obtained hourly data on carbon monoxide (CO), nitrogen dioxide (NO_2_), ozone (O_3_), sulfur dioxide (SO_2_), and particulate matter of median aerodynamic diameter less than 10 and 2.5 microns (PM_10_, PM_2.5 _respectively). CO, NO_2_, O_3 _and SO_2 _were measured using "reference methods" or "equivalent methods" as designated by the United States Environmental Protection Agency [7]. CO was measured using non-dispersive infrared spectrometry, NO_2 _using chemiluminesence, O_3 _using chemiluminesence/ultraviolet photometry and SO_2 _using coulometry/ultraviolet fluorescence. PM_2.5 _and PM_10 _were measured using tapered element oscillating microbalance instruments. When data were available from more than one monitoring site in a city, they were averaged as a measure of average community exposure in each city (see additional file 1: monitoring sites and city area). Values were not imputed for missing data.

Administrative ED visit data are not routinely captured in electronic form from all provinces. Two provinces in this study (Alberta and Ontario) collect some administrative data; however, most ED data are housed in individual institutions [8]. We therefore obtained data from individual institutions which had created their own electronic databases as follows: Montreal (Sir Mortimer B. Davis Jewish General Hospital); Ottawa (Ottawa Civic Hospital); Edmonton (Royal Alexandra Hospital, Stollery Children's Health Centre, Sturgeon Community Hospital and Health Centre, University of Alberta Hospital, Grey Nuns Community Hospital and Health Centre, Misericordia Community Hospital and Health Centre); Saint John (Saint John Regional Hospital, St. Joseph's Hospital); Halifax (Queen Elizabeth II Health Centre); Toronto (St. Michael's Hospital, Sunnybrook Hospital); and Vancouver (St. Paul's Hospital). There is no children's hospital in Saint John, so all visits by children were included in these data. In the case of Edmonton, data from the children's hospital were included in our data set. The other centers have children's hospitals; however, they were not included in our data.

Emergency visit data were coded for the discharge diagnosis using ICD-9 or 10 by medical records staff at each institution. In addition, data pertaining to demographics (e.g. age and sex) and administrative details (e.g. date and time of visit) were obtained for every ED visit. All visits at each institution were included; they were not restricted to individuals who resided in each city. We defined diagnosis groups as follows (ICD-9 and -10 respectively): angina/myocardial infarction (410–414; I20-I22, I24-I25); dysrhythmia/conduction disturbance (426, 427; I44–I49); heart failure (428; I50); respiratory infection (464, 466, 480–487; J05.0, J10–J16, J18, J20–J21); asthma (493; J45); and chronic obstructive pulmonary disease (490–492, 494–496; J40–J44, J47, J67). A control series (anemia, epilepsy, selected nerve disorders (eg. trigeminal neuralgia), selected muscle disorders (eg. muscular dystrophy), selected gastrointestinal disorders (eg. appendicitis), chronic liver disease and cirrhosis, selected gall bladder and intestinal conditions, calculus of lower urinary tract, and hyperplasia of the prostate) was also constructed (280–281.9,345, 350–356, 358–359.5,530–534, 540–543, 560–569, 571,572, 574–578, 594, 600; D50–D53, G40–G41, G50–G52, G54, G57, G58, G60, G70, G71.0–G71.3, G72.1–G72.3, K20–K22, K25–K28, K35–K38, K56–K59, K60–K63, K65–K66, K70, K72.1, K72.9, K73, K74, K75.0, K75.1, K76.6, K76.7, K80–K83, K85–K86, K92.0–K92.2, K92.8, K92.9, N21, N40). ICD-10 nomenclature was introduced at various times at different institutions. The study was approved by the individual research ethics boards at each participating institution and the data were transferred to the Health Canada team following de-identification. No patient contact was made and patients could not be traced.

The daily number of ED visits was modelled as a function of the exponential of air pollution concentrations using generalized linear models in S Plus. The error distribution was specified as quasi-Poisson, where the variance is proportional to the expected response, accommodating over/under dispersion relative to Poisson variation. Natural spline functions of time were employed to adjust for seasonal cycles in air pollution and health outcomes. The number of knots of the cubic polynomials that comprise the splines specify the degree of non-linearity in the spline functions. In order to determine optimal temporal adjustments, natural spline smooths were constructed based on knots placed every *n *weeks for *n *= 1 to 52. Optimality of smoothing was judged objectively based on minimization of the Akaike Information Criterion as a measure of goodness of fit, and maximization of the Bartlett's test p-value as a test for white noise. Sensitivity analyses were conducted where associations were statistically significant, employing knots at every 3, 5 and 17 weeks (approximately 17, 10 and 3 knots per year). Indicator functions were also created for day of week and major Canadian holidays (New Year's Day, Good Friday, Victoria Day, St. Jean Baptiste Day, Canada Day, Civic Holiday, Labour Day, Thanksgiving Day, and Christmas Day). In our analysis of sub-daily effects (see below), we also included indicator variables for time of day (eight 3-hour periods per day).

In order to parsimoniously adjust for effects of weather, we chose temperature and relative humidity as the primary meteorological factors associated with visits. Mean daily temperature and relative humidity with a lag of 0, 1 and 2 days were forced into the model using natural spline functions with 4 degrees of freedom for each lag in order to capture potential non-linearity of the exposure-response.

After making city-specific adjustments for temporal cycles and weather as described above, air pollution terms were added. We examined effects on 2 different time scales. In the conventional analysis, we used single day lags of 0–2 days of the daily average for all pollutants. We also examined effects within a day, by considering 3 hour averages of visits versus 3 hour average pollutant concentrations (e.g. 12 a.m.–3 a.m., 3 a.m.–6 a.m., 6 a.m. – 9 a.m. etc.), lagged up to 12 hours before the time of presentation to the ED. For each lag, regression parameter estimates for each pollutant were pooled among the centers using a fixed or random effects model, depending on the occurrence of statistically significant heterogeneity among effect estimates [9]. Pooled estimates are essentially a weighted average, with results from each city assigned a weight inversely proportional to the sum of within and between city variance. Thus, cities with more precise estimates are weighted more heavily in the pooled estimate. Finally, we calculated the percentage change in visits associated with a change in the pollutant concentration equivalent to the average among all cities. Subgroup analyses were conducted by season (warm season = April to September; cold season = October to March).

## Results

Average air pollution concentrations among sites (Table 1) were not widely divergent, with the exception of NO_2 _concentrations which tended to be lower in Saint John, and SO_2 _concentrations, which were higher in Halifax and Saint John. While data for the pollutant gases were available for essentially all days with the exception of Halifax (where data were missing for approximately one third of days), particulate matter data were less consistently available. Continuous PM monitors were in the process of being introduced in the late 1990s at most sites meaning that data were often missing early in the time-series. Ozone was often negatively correlated with the other pollutants, most strongly during the cool season. Moderate to high correlations were observed among the other pollutants (Table 2). Edmonton, for which visit data were available for multiple institutions, accounted for nearly 70% of all visits (Table 3). Since visit data were only available from selected institutions in each city, visit counts are not proportional to population. Respiratory infections, myocardial infarction/angina and asthma were responsible for the largest number of visits in all centers, while COPD, heart failure and dysrhythmia/conduction disturbance accounted for the fewest. Visits for heart failure exhibited the largest percentage of visits by individuals 65 years or older (76–90% among the centers), followed by COPD (49–76%), and myocardial infarction/angina (53–68%). Visits for asthma were associated with the smallest percentage of individuals 65 or older (4–20%). Visits by children (under 16 years) for asthma and respiratory infections were most frequent in Saint John (44 and 40% respectively) where there is no children's hospital, and Edmonton (41 and 47%) where data from the children's hospital were available, compared to the other sites (0–8% and 0–6%).

**Table 1:** Descriptive air pollution statistics by site

Centre (dates)	Pollutant	Number of days	Mean	25^th^ percentile	75^th^ percentile	Standard Deviation	Percent missing
Montreal	CO (ppm)	2191	0.5	0.3	0.6	0.2	0.0

(1/97–12/02)	NO_2 _(ppb)	2191	19.4	14.0	23.5	7.6	0.0

	SO_2 _(ppb)	2191	4.8	2.7	6.1	3.0	0.0

	O_3 _(ppb)	2191	18.3	11.4	23.5	9.5	0.0

	PM_10 _(μg/m^3^)	1092	25.8	15.9	31.9	14.2	50.2

	PM_2.5 _(μg/m^3^)	1938	8.6	4.1	10.9	6.7	11.5

Ottawa	CO (ppm)	3074	0.9	0.5	1.1	0.5	0.0

(4/92–12/00)	NO_2 _(ppb)	3066	18.8	12.7	24.0	8.8	0.3

	SO_2 _(ppb)	3045	3.9	1.7	5.6	3.0	1.0

	O_3 _(ppb)	3049	17.5	11.5	23.0	8.3	0.8

	PM_10 _(μg/m^3^)	361	20.1	12.0	24.1	11.3	88.3

	PM_2.5 _(μg/m^3^)	954	6.7	3.0	8.7	5.2	69.0

Edmonton	CO (ppm)	3652	0.7	0.4	0.8	0.4	0.0

(4/92–3/02)	NO_2 _(ppb)	3652	21.9	14.7	27.6	9.4	0.0

	SO_2 _(ppb)	3616	2.6	1.3	3.5	1.8	1.0

	O_3 _(ppb)	3652	18.6	11.3	25.2	9.3	0.0

	PM_10 _(μg/m^3^)	2813	22.6	13.3	28.3	13.1	23.0

	PM_2.5 _(μg/m^3^)	1440	8.5	4.6	10.9	6.2	60.6

Saint John	CO (ppm)	1225	0.5	0.2	0.8	0.3	10.6

(7/92–3/96)	NO_2 _(ppb)	1363	9.3	5.1	12.3	5.5	0.5

	SO_2 _(ppb)	1369	7.7	2.7	10.6	7.0	0.1

	O_3 _(ppb)	1367	20.1	14.6	24.4	7.5	0.2

	PM_10 _(μg/m^3^)	0					100.0

	PM_2.5 _(μg/m^3^)	0					100.0

Halifax	CO (ppm)	1032	0.5	0.4	0.6	0.2	34.8

(1/99–12/02)	NO_2 _(ppb)	991	17.5	13.6	21.0	5.8	37.4

	SO_2 _(ppb)	1093	10.0	5.6	13.4	6.6	31.0

	O_3 _(ppb)	1096	22.1	16.4	27.1	7.9	30.8

	PM_10 _(μg/m^3^)	0					100.0

	PM_2.5 _(μg/m^3^)	526	9.8	4.7	11.3	9.0	66.8

Toronto	CO (ppm)	1552	1.0	0.7	1.3	0.4	0

(4/99–6/03)	NO_2 _(ppb)	1552	22.7	17.6	27.5	7.6	0

	SO_2 _(ppb)	1552	4.2	2.4	5.4	2.6	0

	O_3 _(ppb)	1552	22.0	14.3	28.6	10.6	0

	PM_10 _(μg/m^3^)	1006	20.7	13.7	25.7	10.3	35.2

	PM_2.5 _(μg/m^3^)	1552	9.1	4.3	11.9	7.1	0

Vancouver	CO (ppm)	1520	0.6	0.4	0.6	0.2	0.0

(1/99–2/03)	NO_2 _(ppb)	1520	18.7	13.6	19.7	4.6	0.0

	SO_2 _(ppb)	1520	2.6	1.4	3.3	1.5	0.0

	O_3 _(ppb)	1520	10.3	8.4	19.3	7.4	0.0

	PM_10 _(μg/m^3^)	1520	13.6	9.0	15.9	4.9	0.0

	PM_2.5 _(μg/m^3^)	1082	6.8	3.5	8.5	3.6	28.8

**Table 2:** Pearson correlations among pollutants by site. Cool season above diagonal and warm season below diagonal.

		CO	NO_2_	SO_2_	O_3_	PM_10_	PM_2.5_
Montreal	CO		0.83	0.61	-0.51	0.68	0.73

	NO_2_	0.71		0.64	-0.41	0.73	0.77

	SO_2_	0.32	0.49		-0.29	0.51	0.67

	O_3_	-0.22	-0.01	0.21		-0.10	-0.39

	PM_10_	0.52	0.70	0.53	0.41		0.70

	PM_2.5_	0.35	0.40	0.36	0.52	0.79	

Ottawa	CO		0.63	0.13	-0.19	0.58	0.45

	NO_2_	0.45		0.25	-0.30	0.89	0.76

	SO_2_	0.15	0.05		-0.21	0.34	0.47

	O_3_	0.17	0.10	0.14		0.16	-0.36

	PM_10_	0.46	0.74	0.35	0.37		

	PM_2.5_	0.33	0.30	0.28	0.39		

Edmonton	CO		0.74	0.42	-0.57	0.49	0.71

	NO_2_	0.62		0.46	-0.52	0.48	0.57

	SO_2_	0.23	0.30		-0.24	0.27	0.28

	O_3_	-0.21	-0.08	-0.08		-0.29	-0.43

	PM_10_	0.41	0.59	0.25	0.17		0.68

	PM_2.5_	0.42	0.51	0.19	0.11	0.81	

Saint John	CO		0.73	0.50	-0.27		

	NO_2_	0.64		0.58	-0.39		

	SO_2_	0.51	0.51		-0.18		

	O_3_	-0.05	0.10	0.15			

Halifax	CO		0.27	0.00	-0.16		0.01

	NO_2_	0.29		0.23	-0.35		0.09

	SO_2_	-0.06	0.29		-0.28		0.01

	O_3_	-0.11	-0.17	-0.19			0.06

	PM_2.5_	0.30	0.27	0.01	0.38		

Toronto	CO		0.47	0.29	-0.28	0.35	0.39

	NO_2_	0.17		0.62	-0.52	0.64	0.66

	SO_2_	0.10	0.66		-0.49	0.54	0.65

	O_3_	-0.17	0.02	0.19		-0.18	-0.34

	PM_10_	-0.03	0.60	0.60	0.58		0.82

	PM_2.5_	0.01	0.47	0.55	0.62	0.91	

Vancouver	CO		0.70	0.71	-0.67	0.72	0.63

	NO_2_	0.73		0.55	-0.57	0.56	0.55

	SO_2_	0.75	0.69		-0.52	0.55	0.49

	O_3_	-0.34	-0.09	-0.24		-0.63	-0.65

	PM_10_	0.49	0.72	0.58	-0.09		0.87

	PM_2.5_	0.39	0.62	0.49	-0.05	0.87	

**Table 3:** Frequency of visits by center and diagnosis group (mean, standard deviation, visits per day)

Center	Myocardial Infarction/Angina	Heart Failure	Dysrhythmia/Conduction Disturbance	Asthma	Chronic Obstructive Pulmonary Disease	Respiratory Infection
Montreal	4,978	3,833	3,943	2,925	2,394	4,761

	(2.3, 1.5)	(1.8, 1.3)	(1.8, 1.4)	(1.3, 1.3)	(1.1, 1.1)	(2.2, 1.7)

Ottawa	12,156	5,115	6,912	5,873	4,764	8,222

	(3.9, 2)	(1.7, 1.3)	(2.2, 1.5)	(1.9, 1.5)	(1.5, 1.3)	(2.7, 2)

Edmonton	35,207	17,115	26,813	62,563	26,527	90,509

	(9.6, 3.8)	(4.7, 2.3)	(7.3, 3.1)	(17.1, 6.2)	(7.3, 4.5)	(24.8, 14.6)

Saint John	2,435	1,312	1,096	4,771	1,761	8,446

	(1.8, 1.4)	(1, 1)	(0.8, 0.9)	(3.5, 2.3)	(1.3, 1.2)	(6.2, 3.9)

Halifax	2,834	1,661	1,643	2,815	1,978	5,321

	(1.8, 1.5)	(1, 1.1)	(1, 1.1)	(1.8, 1.5)	(1.2, 1.2)	(3.4, 2.3)

Toronto	3,194	2,035	2,897	2,652	1,827	3,563

	(3.1, 1.9)	(1.8, 1.5)	(2.5, 1.8)	(2.1, 1.8)	(1.5, 1.4)	(3.1, 2.2)

Vancouver	2,380	1,242	1,856	1,964	1,240	4,323

	(1.6, 1.3)	(0.8, 0.9)	(1.2, 1.1)	(1.3, 1.2)	(0.8, 1)	(2.8, 2)

Total	63,184	32,313	45,160	83,563	40,491	125,145

Associations between daily average air pollution concentrations, lagged 0–2 days, and cardiac and respiratory visits are shown in Tables 4 and 5. Several positive, statistically significant associations were observed with visits for cardiac conditions, while two negative, statistically significant associations were observed. Associations over multiple lags and/or conditions were observed for CO (lag 0 for both angina/myocardial infarction and heart failure), NO_2 _(lag 0 and 1 for angina/myocardial infarction and lag 0 for heart failure) and particulate matter with myocardial infarction/angina (PM_10 _lag 1) and heart failure (PM_10 _and PM_2.5 _lag 0) as well as SO_2 _with myocardial infarction/angina (lag 0 and 1). Few statistically significant associations (positive or negative) were observed for respiratory conditions. However, ozone was associated with both asthma and COPD visits (lag 2). Associations of eight hour maximum ozone concentration with asthma and COPD visits were similar or smaller in magnitude than associations based on 24-hour average concentration (results not shown). No associations were observed with control conditions.

**Table 4:** Percent increase in cardiac visits (95% confidence interval) for specified change in pollutant concentration.

Pollutant	Lag	Mean	Angina/Myocardial Infarction	Heart Failure	Dysrhythmia
CO	0	0.7	2.1 (0.0, 4.2)*	**3.8 (0.7, 6.9)**	-2.7 (-5.8, 0.5)

	1	ppm	1.6 (-0.5, 3.7)	2.8 (-2.1, 8.0)	-4.0 (-10.1, 2.5)

	2		-0.2 (-2.2, 1.8)	1.7 (-2.7, 6.2)	-2.5 (-8.4, 3.8)

NO_2_	0	18.4	**2.6 (0.2, 5.0)**	**4.7 (1.2, 8.4)**	-1.3 (-4.1, 1.5)

	1	ppb	**2.7 (0.2, 5.3)**	2.8 (-1.3, 7.1)	-0.9 (-3.8, 2.1)

	2		0.7 (-1.6, 3.1)	1.9 (-3.1, 7.1)	0.3 (-2.6, 3.3)

O_3_	0	18.4	-3.4 (-7.8, 1.3)	-1.2 (-6.5, 4.5)	-1.7 (-6.6, 3.4)

	1	ppb	*-3.0 (-5.9, 0.0)†*	-0.5 (-4.4, 3.6)	2.0 (-3.7, 7.9)

	2		-0.3 (-4.0, 3.5)	2.6 (-1.4, 6.7)	1.5 (-4.7, 8.2)

PM_10_	0	20.6	0.0 (-2.1, 2.2)	**9.3 (0.8, 18.4)**	-0.1 (-5.9, 6.1)

	1	μg/m^3^	1.9 (-0.3, 4.1)	0.4 (-2.8, 3.6)	-0.9 (-3.4, 1.6)

	2		-0.3 (-2.5, 1.8)	-0.1 (-3.2, 3.0)	2.1 (-4.0, 8.5)

PM_2.5_	0	8.2	2.0 (-1.3, 5.5)	**6.5 (0.1, 13.4)**	-0.8 (-3.1, 1.5)

	1	μg/m^3^	1.1 (-2.0, 4.3)	-0.1 (-2.8, 2.7)	0.0 (-2.3, 2.3)

	2		0.9 (-1.0, 2.8)	-0.4 (-3.0, 2.3)	0.0 (-2.3, 2.3)

SO_2_	0	5.1	1.7 (-0.2, 3.5)	0.9 (-2.8, 4.7)	-0.7 (-3.1, 1.7)

	1	ppb	**2.1 (0.2, 4.0)**	1.5 (-1.0, 4.1)	0.4 (-3.3, 4.3)

	2		0.0 (-2.3, 2.4)	0.8 (-1.6, 3.3)	*-2.6 (-4.8, -0.3)*

*Positive, statistically significant associations are shown in bold. †Negative, statistically significant associations are shown in italics.

**Table 5:** Percent increase in respiratory visits (95% confidence interval) for specified change in pollutant concentration.

Pollutant	Lag	Mean	Asthma	Chronic Obstructive Pulmonary Disease	Respiratory Infection
CO	0	0.7	-1.8 (-5.8, 2.2)	2.2 (-3.4, 8.1)	-0.8 (-3.2, 1.7)

	1	ppm	-0.3 (-2.3, 1.8)	*-3.3 (-6.1, -0.4)†*	0.7 (-3.0, 4.5)

	2		0.6 (-1.5, 2.7)	-2.3 (-6.1, 1.6)	2.4 (-1.7, 6.7)

NO_2_	0	18.4	-0.4 (-4.4, 3.9)	0.1 (-5.6, 6.2)	-0.9 (-2.9, 1.1)

	1	ppb	-1.2 (-4.6, 2.3)	*-3.4 (-6.6, -0.1)*	0.7 (-3.7, 5.3)

	2		0.0 (-2.4, 2.5)	-4.8 (-11.5, 2.5)	0.6 (-1.4, 2.6)

O_3_	0	18.4	0.1 (-3.8, 4.2)	-2.2 (-5.9, 1.7)	-0.6 (-3.1, 1.9)

	1	ppb	4.3 (-1.3, 10.2)	2.2 (-1.7, 6.3)	0.1 (-2.3, 2.5)

	2		3.2 (0.3, 6.2)*	3.7 (-0.5, 7.9)	-0.1 (-3.0, 2.9)

PM_10_	0	20.6	5.3 (-2.2, 13.4)	-0.6 (-3.3, 2.2)	0.0 (-1.8, 1.8)

	1	μg/m^3^	0.9 (-1.1, 3.0)	-1.7 (-7.6, 4.5)	-0.9 (-2.7, 0.9)

	2		1.1 (-2.4, 4.8)	-5.3 (-11.4, 1.2)	-1.2 (-3.0, 0.6)

PM_2.5_	0	8.2	1.7 (-2.5, 6.1)	-1.8 (-6.1, 2.7)	0.6 (-1.4, 2.7)

	1	μg/m^3^	0.9 (-1.1, 3.0)	-0.9 (-3.6, 1.8)	0.6 (-1.1, 2.4)

	2		0.4 (-1.6, 2.4)	-0.2 (-5.0, 4.8)	-0.2 (-1.9, 1.5)

SO_2_	0	5.1	-1.1 (-2.9, 0.7)	-1.9 (-4.3, 0.6)	-0.6 (-2.1, 0.9)

	1	ppb	-1.8 (-4.2, 0.7)	-1.4 (-4.4, 1.8)	-0.2 (-1.7, 1.3)

	2		-1.1 (-2.8, 0.7)	-3.0 (-6.7, 0.9)	0.6 (-1.3, 2.6)

*Positive, statistically significant associations are shown in bold. †Negative, statistically significant associations are shown in italics.

**Figure 1:**
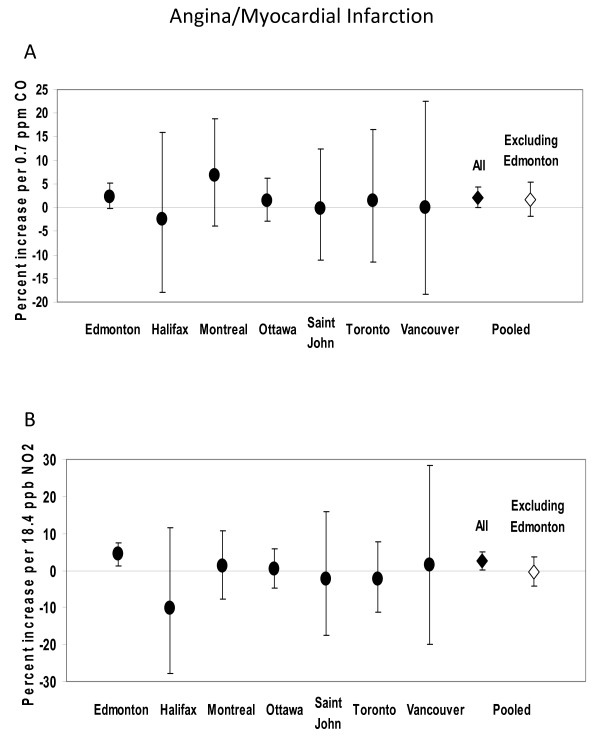
**Percent increase in emergency department visits for angina/myocardial infarction by center.** (Point estimates and 95% confidence intervals are shown, for CO (panel A) and NO2 (panel B)).

**Figure 2:**
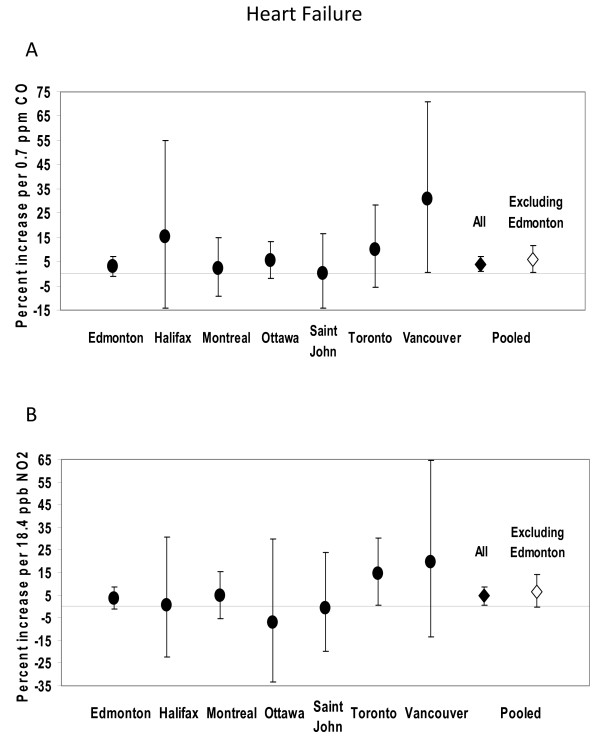
**Percent increase in emergency department visits for heart failure by center**. (Point estimates and 95% confidence intervals are shown, for CO (panel A) and NO2 (panel B)).

**Figure 3:**
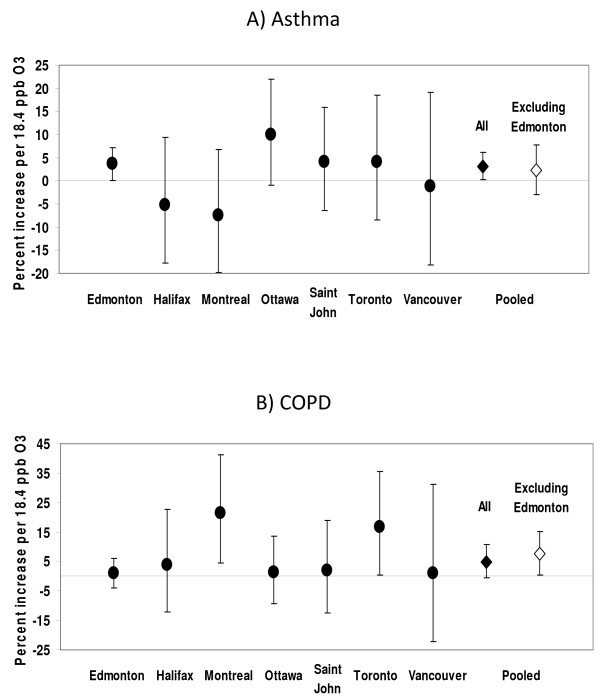
**Percent increase in respiratory emergency department visits by center**. (Point estimates and 95% confidence intervals are shown for asthma (panel A) and COPD (panel B)).

Effects of CO and NO_2 _on myocardial infarction/angina and heart failure by city are shown in Figures 1 and 2. Pooled estimates are shown both with and without Edmonton in order to examine the consistency of effects relative to the center with the largest sample size and hence most precise estimates assigned the greatest weight in calculating the pooled estimate. In the case of myocardial infarction/angina, effect sizes in Edmonton and Ottawa were similar in magnitude, while effect sizes in other centers were more variable and less precise. With respect to heart failure, effects in Edmonton and Montreal were closer in magnitude and those elsewhere tended to be more variable and less precise. The effects of ozone on asthma and COPD visits by city are shown in Figure 3. Effect size estimates were consistently less precise in centers other than Edmonton. With the exception of the association between NO_2 _and myocardial infarction/angina visits, pooled estimates were generally similar in magnitude when Edmonton was excluded, and somewhat less precise. This indicates both that the pooled estimate is not singularly reflecting the estimate from Edmonton and that the pooled estimate can be statistically significant even when effects in individual centers are not, because of the collective power of the pooled sample. There was no evidence of heterogeneity among centers (p > 0.05 for Q-statistic).

**Figure 4:**
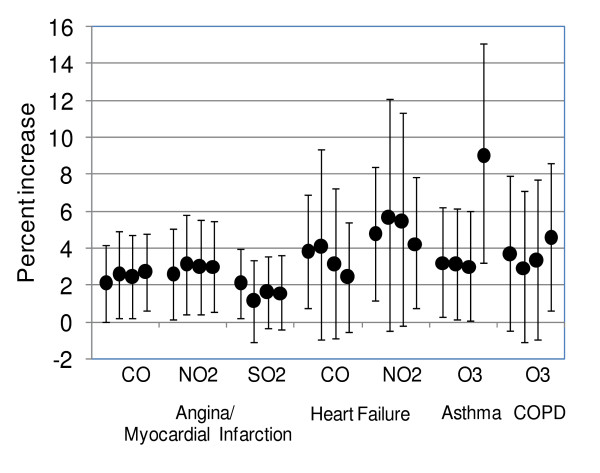
**Percent increase in emergency department visits for selected pollutants, diagnostic groups per mean pollutant concentration**. (Point estimates and 95% confidence intervals are shown by number of knots in natural spline functions of time; from left to right, base analysis where number of knots is optimized separately in each city, and knots every 3, 5 and 17 weeks).

Associations of CO and NO_2 _with myocardial infarction/angina and heart failure tended to be of larger magnitude during the warm season. CO (lag 0) was associated with a 5.2% increase in myocardial infarction/angina visits (95% CI, 0.4–10.2) per 0.7 ppm, and a 6.8% increase in heart failure visits (95% CI, -0.8–14.9), which were approximately twice the magnitude of the effects seen over the whole year. Similarly, NO_2 _was associated with a 4.0% increase in myocardial infarction/angina visits (95% CI, -0.5–8.8) per 18.4 ppb, and a 7.2% increase in heart failure visits (95% CI, 0.5–14.4). These effects were approximately 50% larger than those seen over the whole year. Effects of PM_2.5 _and SO_2 _on myocardial infarction/angina were weaker in the individual seasons compared to the analysis over the entire year. The magnitude of the association between ozone and COPD visits was nearly twice as large during the warm season as over the whole year (6.2% increase in visits, 95% CI, 0.1–12.7, per 18.4 ppb) and the associations of PM_10 _and PM_2.5 _with asthma visits were respectively nearly three- and over fourfold larger (14.4% increase in visits, 95% CI, 0.2–30.7, per 20.6 μg/m^3 ^PM_10 _and 7.6% increase in visits, 95% CI, 5.1–10.1, per 8.2 μg/m^3 ^PM_2.5_). No consistent associations were observed between any pollutants and cardiac or respiratory visits during the winter. As shown in Figure 4, the magnitude of effects was generally not sensitive to the number of knots in the natural spline, but associations of SO_2 _with MI/angina and of CO and NO_2 _with heart failure were only statistically significant in the base analysis where the number of knots in the natural spline was optimized separately in each city (see additional file 2: natural spline specifications). The effect of ozone on asthma was much larger with knots at every 17 weeks. In a two pollutant model of MI/angina visits, including CO and NO_2_, the effects of both pollutants were reduced in magnitude and statistical significance. At their mean concentrations, CO was associated with a 0.16% increase in visits (95% CI, -0.60–0.92%, p = 0.68) and NO_2 _was associated with a 1.18% increase in visits (95% CI, -2.64–5.15%, p = 0.55). There was no evidence of consistent associations between any pollutant and cardiac or respiratory visits on sub-daily time scales (see additional files 3 and 4: 3 hour results cardiac and 3 hour results respiratory).

## Discussion

We have identified significant effects of air quality on acute cardiac and respiratory presentations in a large, multi-center Canadian study of air pollution and ED visits. Unique features of this study include application of a consistent methodology to, and pooling of results from multiple diverse sites, each with over 10,000 visits, with a total of nearly 400,000 visits; examination of a comprehensive suite of cardiac and respiratory conditions; evaluation of effects over multiple time scales; and assessment of effects at generally lower pollutant concentrations than reported elsewhere. With the exception of a study in Atlanta of approximately 1 million visits for cardiovascular and respiratory disease [10-12], many other recent studies have been based on fewer than 10,000 visits, and have examined single conditions or were restricted to specific age groups or seasons.

Compared to other recent studies in Canada of the same sites, our present results confirm our earlier findings in Saint John of associations between ozone and asthma and COPD visits [13,14]. In contrast, we found no consistent associations in the current study with respiratory infections, despite comprising the largest single diagnostic category. We found significant associations, however, between cardiac visits and CO and NO_2_, which were not detected in Saint John. As a small city, Saint John does not have large traffic volumes, so it is characterized by low ambient concentrations of these pollutants. Villeneuve et al. [15] also reported associations of CO and NO_2 _with ED visits for stroke and transient ischemic attacks. Conversely, Saint John has relatively high concentrations of SO_2_. There was some evidence of an association between SO_2 _and angina/myocardial infarction in the current analysis, but not with any of the respiratory diagnoses.

Elsewhere, several recent studies have evaluated associations between air pollution and ED visits for respiratory disease. CO exhibited an association with COPD [11,16] and upper respiratory infection (URI) visits [11], as well as all respiratory visits [12]. Associations were observed between NO_2 _and visits for asthma [17,18], COPD [11], URI [11] and lower respiratory disease [19], as well as all respiratory visits [12]. Ozone was associated with respiratory visits in several studies, including visits for URI [11], asthma [18,20,21], COPD [16], and all respiratory visits [12]. Associations with particulate matter were observed for asthma [18,20], URI and pneumonia [11], as well as all respiratory visits [12]. Finally, SO_2 _exhibited associations with asthma visits [18,20,21]. A study of CO, PM_1_, PM_2.5 _and PM_10 _relative to respiratory visits in Spokane, revealed generally weak and inconsistent associations [22]. The authors also examined associations with hospital admissions and mortality and again results were inconsistent. Fewer studies have examined associations between air pollution and visits for cardiovascular disease. In a study in Atlanta, CO, NO_2 _and PM_2.5 _(and specific components) were associated with visits for cardiovascular disease [10,12]. CO, NO_2_, SO_2 _and PM_2.5 _were associated with visits for cardiovascular disease in those 65 years of age or older in Sydney [23]. Only CO was associated with visits for angina or myocardial infarction in a study in Sao Paulo [24].

We observed associations between daily average air pollution concentrations and emergency department visits with lags of zero to two days, but not for three hour average concentrations lagged up to 12 hours within the same day. We previously reported that approximately 30% and 70% respectively of those visiting the emergency department with respiratory or cardiac conditions did so within less than one day of developing the symptoms most responsible for their visit [5]. This is consistent with our current findings of predominant effects of CO and NO_2 _on cardiac conditions at lag 0, and of O_3 _on respiratory conditions at lag 2. In contrast, Villeneuve et al. [25] reported significant associations between 6 hour average weather variables such as thunderstorm activity, and visits by children for asthma.

Somewhat larger effects were observed for visits for heart failure compared to myocardial infarction. Individuals with heart failure have been identified as one group at particular risk of air pollution related mortality [26]. Of respiratory conditions, effects of ozone were larger for COPD compared to asthma visits, particularly in the warm season. Individuals with COPD could be considered to have a similar relative degree of frailty compared to those with asthma, as those with heart failure compared to myocardial infarction/angina. Indeed, in all centers in the current analysis, those making visits for COPD were, not surprisingly, older than those with asthma, just as those presenting with heart failure were older than those with myocardial infarction/angina.

We also observed larger effects in the warm season, which is consistent with knowledge of time-activity patterns [27]. These data indicate that individuals spend more time outdoors during warm weather, increasing their exposure to outdoor air pollution and by consequence reducing exposure misclassification of exposures derived from fixed site monitoring stations. This lends additional credibility to the existence of a causal association between exposure and response.

While the pathophysiology of the impacts of air pollution on the respiratory system has been well documented over many years, particularly in relation to ozone and particulate matter, mechanisms of action on the cardiovascular system have received more recent attention. These include effects on arterial vasoconstriction [28], heart rate variability [29,30], blood clotting [31] and formation of atherosclerotic plaques [32]. Several studies have documented an increased frequency of discharges of implantable defibrillators related to short-term increases in exposure to particulate matter, CO and NO_2 _[33,34]. Peters et al. [35] recently reported an association between exposure to traffic and onset of myocardial infarction.

We relied on diagnostic information provided in administrative data records, which would be expected to be associated with a degree of misclassification. However, in an earlier evaluation of emergency department diagnostic classification for respiratory and cardiac conditions, we observed substantial or better interobserver agreement in diagnostic classification (κ = 0.69 to 0.84 for the categories considered here), respiratory infections notwithstanding (κ = 0.53), and there was no evidence of diagnostic bias in relation to daily air pollution level [36]. Although compared to many recent studies, we had the advantage of a relatively large sample size, our ability to detect effects of particulate matter may have been constrained by the limited availability of data in some centers. Applying a consistent methodology to multiple sites is also advantageous, but one center (Edmonton) had a disproportionately large sample. Nonetheless, sample sizes from other sites, or for specific conditions pooled over multiple sites were still larger than most recent studies of ED visits, and pooled estimates were generally similar, although less precise, when Edmonton was excluded. Since, with the exception of Edmonton and Saint John, data were available only for selected hospitals, these may not be representative of the entire population in a given city. However, in the context of Canada's publicly funded universal healthcare system, large differences between hospitals are less likely. Statistically significant heterogeneity among sites was not detected, probably because of the large within versus between site variance of effects. While we have attempted to focus on effects which exhibited consistency over multiple lags and/or diagnosis groups, we have nonetheless conducted numerous hypothesis tests, increasing the number of potentially false positive results. However, the number of positive, statistically significant associations with visits for cardiac conditions exceeded the number which could be expected by chance alone, and few negative and significant associations were observed.

## Conclusion

We conducted a time series analysis of air pollution and cardiac and respiratory emergency department visits in seven Canadian cities, based on nearly 400,000 visits to 14 hospitals. CO and NO_2 _exhibited the most consistent associations with cardiac conditions, while ozone was most consistently associated with respiratory visits. PM_10 _and PM_2.5 _were strongly associated with asthma visits during the warm season. No consistent associations were observed between three hour average counts of cardiac or respiratory visits and three hour average pollutant concentrations lagged up to 12 hours. In addition to their consistency with other results, these results add further weight to arguments regarding the role of air pollution in contributing to adverse health events, and imply that interventions to reduce these pollutants are warranted in an effort to reduce cardio-respiratory ED visits.

## Abbreviations

CO: carbon monoxide; COPD: chronic obstructive pulmonary disease; ED: emergency department; ICD: international classification of diseases; MI: myocardial infarction; NAPS: national air pollution surveillance; NO_2_: nitrogen dioxide; O_3_: ozone; PM_1_: particulate matter of median aerodynamic diameter less than 1 μm; PM_2.5_: particulate matter of median aerodynamic diameter less than 2.5 μm; PM_10_: particulate matter of median aerodynamic diameter less than 10 μm; ppb: parts per billion; ppm: parts per million; SO_2_: sulfur dioxide; URI: upper respiratory infection.

## Competing interests

The authors declare that they have no competing interests.

## Authors' contributions

DMS conceived of the study, directed the statistical analysis and drafted the manuscript. MS conducted the statistical analysis. BHR participated in study design and directed data acquisition. JAL participated in study design and contributed data. All authors contributed to drafting the manuscript and read and approved the final version.

## Supplementary Material

Additional file 1 Number of monitors by city and pollutant and city surface area. Click here for file

Additional file 2 **Knots were placed every n weeks in the natural spline function of time.** n is shown by diagnosis group and site. Click here for file

Additional file 3 **Percent increase in cardiac visits by pollutant, lag and diagnosis, for change in pollutant concentration equal to mean among all centres.** Analysis is based on 3 hour average pollutant concentrations and emergency visits. Effect estimates are pooled among centres. Click here for file

Additional file 4 **Percent increase in respiratory visits by pollutant, lag and diagnosis, for change in pollutant concentration equal to mean among all centres.** Analysis is based on 3 hour average pollutant concentrations and emergency visits. Effect estimates are pooled among centres. Click here for file
