# Supplementary material for: Air pollution and emergency department visits for cardiac and respiratory conditions: a multi-city time-series analysis
Source: Environ Health. 2009 Jun 10;8:25. doi: 10.1186/1476-069X-8-25 (PMC2703622; doi:10.1186/1476-069X-8-25)
Supplement: Additional file 1 — Number of monitors by city and pollutant and city surface area. [file 1476-069X-8-25-S1.pdf]

Number of monitors by city and pollutant and city surface area

City	Number of monitors						City surface area* (km^2)
	CO	NO2	O3	SO2	PM10	PM25	
Montreal	7	13	14	6	2	7	500
Ottawa	2	2	2	2	1	1	2778
Edmonton	3	3	3	3	2	3	684
Halifax	1	1	1	1	0	1	5496
Saint John	1	2	2	2	0	0	1462
Toronto	6	9	9	6	4	7	629
Vancouver	11	14	16	11	9	1	2879

*based on census boundaries.
