# Supplementary material for: Air pollution and emergency department visits for cardiac and respiratory conditions: a multi-city time-series analysis
Source: Environ Health. 2009 Jun 10;8:25. doi: 10.1186/1476-069X-8-25 (PMC2703622; doi:10.1186/1476-069X-8-25)
Supplement: Additional file 2 — Knots were placed every n weeks in the natural spline function of time. n is shown by diagnosis group and site. [file 1476-069X-8-25-S2.pdf]

Knots were placed every n weeks in the natural spline function of time. n is shown by diagnosis group and site.

	Montreal	Ottawa 1992-1999	Ottawa 2000*	Edmonton	Saint John	Halifax	Toronto - St. Michael's	Toronto - Sunnybrook	Vancouver
Angina/ Myocardial Infarction	13	23	3	9	5	3	17	7	15
Heart Failure	25	21	12	24	6	9	8	10	21
Dysrhythmia	14	9	3	23	9	5	24	7	9
Asthma	5	5	2	2	4	4	11	7	12
Chronic Obstructive Pulmonary Disease	8	7	10	4	12	6	7	5	7
Respiratory Infection	8	7	5	2	3	4	5	5	12

*Visit data were missing for the period September-December 1999; thus data from 2000 were treated as a separate series.
