# Supplementary material for: Air pollution and emergency department visits for cardiac and respiratory conditions: a multi-city time-series analysis
Source: Environ Health. 2009 Jun 10;8:25. doi: 10.1186/1476-069X-8-25 (PMC2703622; doi:10.1186/1476-069X-8-25)
Supplement: Additional file 3 — Percent increase in cardiac visits by pollutant, lag and diagnosis, for change in pollutant concentration equal to mean among all centres. Analysis is based on 3 hour average pollutant concentrations and emergency visits. Effect estimates are pooled among centres. [file 1476-069X-8-25-S3.pdf]

Percent increase in cardiac visits by pollutant, lag and diagnosis, for change in pollutant concentration equal to mean among all centres. Analysis is based on 3 hour average pollutant concentrations and emergency visits. Effect estimates are pooled among centres

			Myocardial Infarction/ Angina		Heart Failure		Dysrhythmia/ Conduction Disturbance	
Pollutant	Lag (x 3 hours)	Mean pollutant concentration	Percent increase	T*	Percent increase	T	Percent increase	T
CO	0	0.7	8.37	4.48	4.40	1.26	5.13	2.30
	1	ppm	5.55	1.53	0.57	0.14	-0.92	-0.23
	2		-3.37	-2.19	-1.63	-0.76	-8.71	-4.10
	3		-9.70	-4.64	-3.50	-1.63	-11.31	-4.17
	4		-5.97	-2.39	-3.81	-1.78	-8.43	-2.35
	5		-4.83	-3.19	0.06	0.01	-4.85	-1.42
NO_2	0	18.3	8.34	4.67	7.59	3.04	5.22	2.20
	1	ppb	7.65	2.09	4.44	1.36	4.16	1.74
	2		-4.98	-1.49	-5.57	-1.39	-10.40	-3.56
	3		-10.90	-3.08	-5.12	-2.17	-12.09	-3.37
	4		-8.20	-5.06	-1.70	-0.50	-7.48	-3.46
	5		0.10	0.06	2.33	0.57	-0.35	-0.11
O_3	0	19.8	-1.66	-0.83	-3.00	-1.18	-2.34	-0.66
	1	ppb	-3.13	-1.47	-3.58	-0.99	0.88	0.15
	2		6.13	2.61	3.80	0.75	10.71	2.33
	3		8.47	3.71	-0.18	-0.05	5.84	1.39
	4		0.30	0.10	-5.73	-2.87	-1.89	-0.47
	5		-4.17	-1.20	-7.77	-3.50	-4.66	-1.21
PM_{10}	0	22.3	1.53	0.18	4.28	0.37	19.18	2.07
	1	$\mu g/m^3$	0.27	0.03	-14.83	-1.37	9.55	1.04
	2		7.71	0.87	-7.55	-0.65	7.42	0.81
	3		2.35	0.27	-15.68	-1.40	-1.83	-0.21
	4		-6.20	-0.74	1.53	0.13	-6.91	-0.77

	5		3.33	0.38	0.09	0.01	-3.85	-0.42
$PM_{2.5}$	0	8.5	2.06	0.74	-1.08	-0.35	3.16	1.20
	1	$\mu g/m^3$	2.01	0.85	-2.74	-0.87	3.52	0.65
	2		-1.39	-0.43	0.24	0.08	3.27	1.03
	3		-1.34	-0.55	0.89	0.27	2.59	0.83
	4		0.03	0.01	-1.51	-0.50	1.74	0.53
	5		-0.48	-0.14	1.02	0.36	1.94	0.76
SO_2	0	5.5	0.35	0.31	0.12	0.10	0.12	0.10
	1	ppb	0.13	0.15	0.18	0.16	-0.52	-0.43
	2		-0.93	-0.83	-0.46	-0.38	-1.43	-1.04
	3		-0.10	-0.11	-0.15	-0.12	-1.41	-0.95
	4		0.37	0.29	0.31	0.25	0.98	0.79
	5		0.77	0.86	-0.74	-0.61	-0.29	-0.17

*T-ratio=β/standard error(β); $|T| > 1.96$ indicates statistical significance corresponding to $p < 0.05$
