# Supplementary material for: Air pollution and emergency department visits for cardiac and respiratory conditions: a multi-city time-series analysis
Source: Environ Health. 2009 Jun 10;8:25. doi: 10.1186/1476-069X-8-25 (PMC2703622; doi:10.1186/1476-069X-8-25)
Supplement: Additional file 4 — Percent increase in respiratory visits by pollutant, lag and diagnosis, for change in pollutant concentration equal to mean among all centres. Analysis is based on 3 hour average pollutant concentrations and emergency visits. Effect estimates are pooled among centres. [file 1476-069X-8-25-S4.pdf]

Percent increase in respiratory visits by pollutant, lag and diagnosis, for change in pollutant concentration equal to mean among all centres. Analysis is based on 3 hour average pollutant concentrations and emergency visits. Effect estimates are pooled among centres

			Asthma		Chronic Obstructive Pulmonary Disease		Respiratory Infection	
Pollutant	Lag (x 3 hours)	Mean pollutant concentration	Percent increase	T*	Percent increase	T	Percent increase	T
CO	0	0.7	4.17	2.45	7.96	2.32	3.28	1.12
	1	ppm	6.88	1.75	5.46	1.58	4.58	1.28
	2		1.87	0.61	-6.04	-1.64	-0.70	-0.51
	3		-0.76	-0.39	-6.08	-1.37	-1.02	-0.20
	4		3.22	0.63	-2.87	-0.63	1.53	0.29
	5		1.13	0.23	-3.68	-1.39	-0.24	-0.05
NO_2	0	18.3	4.92	1.25	6.43	1.86	2.36	1.41
	1	ppb	4.41	0.84	6.57	2.22	4.66	1.55
	2		-2.83	-1.05	-6.45	-1.16	-2.00	-0.56
	3		-6.75	-1.99	-8.91	-1.65	-6.28	-1.08
	4		-4.38	-0.92	-4.79	-1.35	-2.48	-0.85
	5		-4.32	-1.18	-0.59	-0.18	-1.68	-0.72
O_3	0	19.8	-4.56	-1.36	2.20	0.87	-1.16	-0.67
	1	ppb	-3.45	-1.07	-0.75	-0.30	-2.48	-0.75
	2		5.02	1.52	8.97	1.97	7.14	1.56
	3		6.80	2.19	5.62	1.21	6.62	1.91
	4		-2.79	-1.19	-5.05	-2.35	-2.58	-1.28
	5		-3.84	-1.08	-7.40	-3.58	-5.16	-1.45
PM_{10}	0	22.3	9.75	1.36	10.31	1.08	-3.16	-0.45
	1	$\mu g/m^3$	6.56	0.90	14.80	1.49	-13.01	-1.89
	2		6.82	0.93	12.80	1.27	-12.78	-1.84
	3		4.75	0.66	12.12	1.21	-8.82	-1.26
	4		13.00	1.77	5.57	0.57	-4.64	-0.65

	5		12.57	1.70	7.95	0.79	-3.20	-0.44
$PM_{2.5}$	0	8.5	5.94	1.48	-0.44	-0.15	-0.24	-0.10
	1	$\mu g/m^3$	2.92	0.91	-2.76	-0.60	-3.00	-0.70
	2		1.49	0.28	-6.03	-0.91	-2.54	-0.55
	3		-1.11	-0.23	-2.34	-0.57	-0.24	-0.06
	4		-1.89	-0.34	-2.67	-0.82	2.03	0.46
	5		-4.61	-0.98	-2.69	-0.81	-0.02	0.00
SO_2	0	5.5	-1.27	-1.62	-0.64	-0.59	-0.78	-1.32
	1	ppb	0.37	0.21	1.34	0.92	-0.91	-1.53
	2		-0.57	-0.74	-1.35	-0.90	0.37	0.63
	3		0.31	0.35	-2.07	-1.78	-0.78	-1.28
	4		-1.51	-1.88	-1.31	-0.80	-0.04	-0.03
	5		-0.17	-0.11	-0.36	-0.23	0.16	0.17

*T-ratio=β/standard error(β); $|T| > 1.96$ indicates statistical significance corresponding to $p < 0.05$
